# Large Impact of Low Concentration Oxidized LDL on Angiogenic Potential of Human Endothelial Cells: A Microarray Study

**DOI:** 10.1371/journal.pone.0047421

**Published:** 2012-10-24

**Authors:** Magomed Khaidakov, Sona Mitra, Xianwei Wang, Zufeng Ding, Nalini Bora, Valery Lyzogubov, Francesco Romeo, Steven A. Schichman, Jawahar L. Mehta

**Affiliations:** 1 Central Arkansas Healthcare System and the University of Arkansas for Medical Sciences, Little Rock, Arkansas, United States of America; 2 Dipartimento di Medicina Interna, Università di Roma Tor Vergata, Roma, Italy; University of California San Francisco, United States of America

## Abstract

Oxidized LDL (ox-LDL) is a key factor in atherogenesis. It is taken up by endothelial cells primarily by ox-LDL receptor-1 (LOX-1). To elucidate transcriptional responses, we performed microarray analysis on human coronary artery endothelial cells (HCAECs) exposed to small physiologic concentration of ox-LDL- 5 µg/ml for 2 and 12 hours. At 12 hours, cultures treated with ox-LDL exhibited broad shifts in transcriptional activity involving almost 1500 genes (>1.5 fold difference, p<0.05). Resulting transcriptome was enriched for genes associated with cell adhesion (p<0.002), angiogenesis (p<0.0002) and migration (p<0.006). Quantitative PCR analysis revealed that LOX-1 expression in HCAECs is at least an order of magnitude greater than the expression of other major ox-LDL specific receptors CD36 and MSR1. In keeping with the data on LOX-1 expression, pre-treatment of HCAECs with LOX-1 neutralizing antibody resulted in across-the-board inhibition of cellular response to ox-LDL. Ox-LDL upregulated a number of pro-angiogenic genes including multiple receptors, ligands and transcription factors and altered the expression of a number of genes implicated in both stimulation and inhibition of apoptosis. From a functional standpoint, physiologic concentrations of ox-LDL stimulated tube formation and inhibited susceptibility to apoptosis in HCAECs. In addition, ox-LDL exposure resulted in upregulation of miR-1974, miR-1978 and miR-21 accompanied with significant over-presentation of their target genes in the downregulated portion of ox-LDL transcriptome. Our observations indicate that ox-LDL at physiologic concentrations induces broad transcriptional responses which are mediated by LOX-1, and are, in part, shaped by ox-LDL-dependent miRNAs. We also suggest that angiogenic effects of ox-LDL are partially based on upregulation of several receptors that render cells hypersensitive to angiogenic stimuli.

## Introduction

Studies in the past decade have identified oxidation of low density lipoproteins (LDL) as a primary factor triggering atherogenesis [1]. Oxidative modification of LDL constituents brings about a fundamental shift in its destination. Oxidized-LDL (ox-LDL) is poorly recognized by LDL receptors and, instead, becomes a ligand for scavenger receptors which re-route ox-LDL from liver to peripheral tissues including vascular wall.

In endothelial cells, ox-LDL is captured primarily by ox-LDL receptor-1 (LOX-1). Various oxidatively modified components of internalized ox-LDL particle generate complex signaling cascades resulting in endogenous production of reactive oxygen species (ROS), endothelial dysfunction, recruitment (chemotaxis and adhesion) and trans-endothelial migration of monocytes with consequent transformation into foam cells and proliferation of vascular smooth muscle cells [1]–[3].

Previous microarray studies utilized moderate to high ox-LDL concentrations [4], [5] which are cytotoxic, pro-apoptotic and anti-angiogenic [6]–[8]. The observed transcriptional changes in response to cytotoxic concentrations are likely to comprise a mixture of mutually exclusive signaling sequences which are difficult to interpret in terms of contribution of ox-LDL to the process of atherogenesis. Therefore, in order to evaluate physiological effects of ox-LDL we analyzed transcriptional responses of human coronary artery endothelial cells (HCAECs) to ox-LDL at a concentration within the range shown to be non-toxic for endothelial cells.

## Results

### Low Concentration ox-LDL Induces Broad Transcriptional Shifts

We have previously shown that concentrations ox-LDL <10 µg/ml are non-toxic to the endothelial cells [9]. In the present studies, exposure of HCAECs to 5 µg/ml ox-LDL for 2 hours did not produce significant changes in the transcriptional profile. After 12 hours of exposure, however, the expression of close to 1500 genes (Table S1) changed significantly (cutoff value of ≥1.5-fold, p<0.05). More stringent selection (≥2-fold, p<0.01) yielded 596 genes with 221 of them downregulated and 375 upregulated (Table S2).

The pathway analysis [10] of differentially expressed genes set is shown in Table 1. In terms of the pathways mechanistically linked to atherogenesis, differential analysis revealed enrichment for genes involved in protein kinase cascade (p<0.004), cell adhesion (p<0.002), angiogenesis (p<0.0002), regulation of cell proliferation (p<0.04) and migration (p<0.006). For further validation studies, we selected genes implicated in angiogenesis and apoptosis.

**Table 1:** EASE pathway analysis of differentially expressed genes.

Term	%	P value
Phosphoprotein	52.5	1.50E−14
Alternative splicing	49.6	4.90E−09
EGF-like, laminin	1.7	5.80E−05
Intracellular signaling cascade	11.7	5.90E−05
Angiogenesis	1.9	1.50E−04
Negative chemotaxis	0.8	1.60E−04
Phospholipid homeostasis	0.8	1.60E−04
Steroid metabolic process	3.1	7.40E−04
Orange subgroup	0.8	7.60E−04
Plexin/semaphorin/integrin	1.5	7.80E−04
TGFb receptor signaling pathway	1.7	8.40E−04
Negative regulation of cell differentiation	3.1	1.40E−03
Lipid synthesis	1.9	2.20E−03
Focal adhesion	3.1	2.20E−03
Regulation of kinase activity	4.2	2.30E−03
Acetylation	17.6	3.00E−03
Protein kinase cascade	4.2	3.40E−03
Response to oxygen levels	2.3	3.50E−03
Regulation of cell proliferation	7.1	3.90E−03
Cell migration	3.3	5.20E−03
Reverse cholesterol transport	0.8	7.40E−03
PDGF receptor signaling pathway	0.8	1.20E−02
Cell adhesion	6.1	1.40E−02

From the ≥1.5 fold differential expression pool, we arbitrarily selected a group of LOX-1 antibody sensitive genes defined as those that reversed to control values by more than ≥30% and conducted separate pathway analyses for upregulated and downregulated sub-groups (Figure S1). Interestingly, in the upregulated portion of thus screened transcriptome we found enrichment themes very similar to those observed from the entire 2-fold differentially expressed gene set including adhesion, angiogenesis, protein kinase cascade and cell motion. On the other hand, downregulated genes showed enrichment for negative regulation of gene expression, cell communication and catalytic activity as well as regulation of division and apoptosis.

### Ox-LDL Induced Transcriptome is LOX-1 Dependent

In microarray studies, scavenger receptors CD36 and MSR1 were expressed in very low amount (baseline) and did not show any appreciable ox-LDL-induced expression. In contrast, there was a significant basal expression of LOX-1, and it increased by about 30% when cells were treated with ox-LDL (p<0.05).

**Figure 1:**
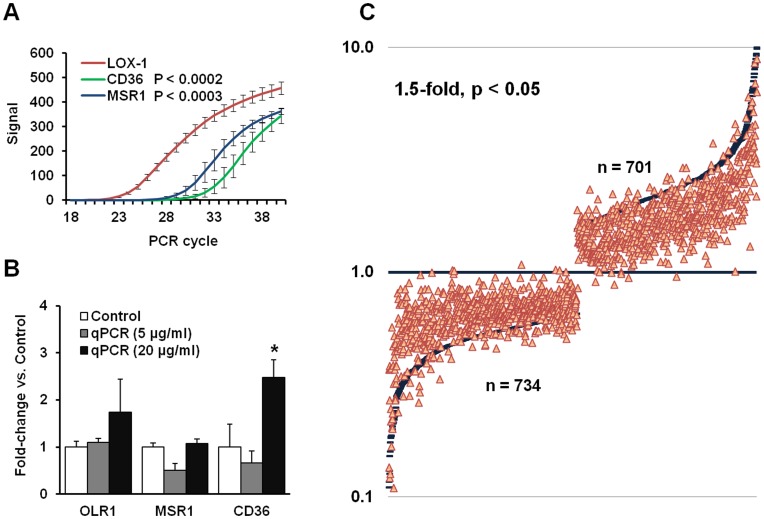
Ox-LDL transcriptome and its dependence on LOX-1 in endothelial cells. (**A**) – basal expression of scavenger receptors in HCAECs evaluated by qPCR. (**B**) – ox-LDL induced expression of scavenger receptors in HCAECs by qPCR. (**C**) - Differentially expressed genes (1.5-fold, p<0.05) in HCAECs exposed to 5 µg/ml ox-LDL for 12 hrs. Blue lines represent genes up- or down-regulated more than 2-fold. Red triangles represent expression of these genes in cultures pre-treated with LOX-1 neutralizing antibody (TS92, 10 mg/ml) before ox-LDL exposure.

To further evaluate relative expression of other major scavenger receptors, we performed qPCR for LOX-1, CD36 and MSR1 in parallel with 12 hour exposure to 5 or 20 µg/ml of ox-LDL. Basal LOX-1 expression was at least 30-fold and 200-fold greater than the expression of MSR1 and CD36, respectively (Figure 1A). Small concentration of ox-LDL (5 µg/ml) tended to downregulate CD36 and MSR1, whereas in response to higher concentration (20 µg/ml), MSR1 did not change and CD36 increased >2-fold (p<0.02, Figure 1B). Compared to control group, LOX-1 was upregulated by 47% in HCAECs exposed to ox-LDL 5 µg/ml and by 67% in HCAECs exposed to ox-LDL 20 µg/ml.

In line with our findings of relative expression of scavenger receptors, pre-treatment of HCAECs with LOX-1 neutralizing antibody (TS92) resulted in almost universal retreat of differentially expressed genes towards control values (Figure 1C).

#### LOX-1 is involved in angiogenesis *in vitro*

**Figure 2:**
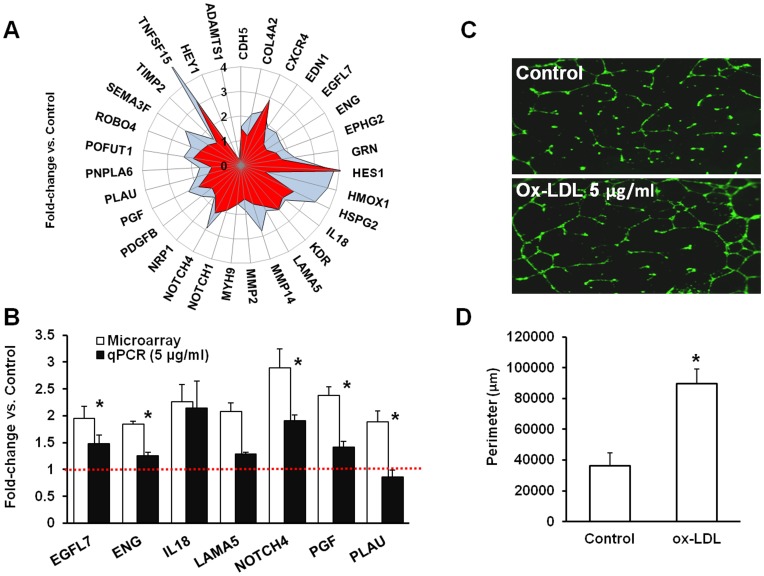
Effects of ox-LDL on genes implicated in angiogenesis. (**A**) - Differentially expressed genes involved in angiogenesis (Blue – exposure to ox-LDL alone; Red- pre-treatment with LOX-1 antibody followed by exposure to ox-LDL; (**B**) **-** validation of microarray data on select genes (white bars) by qPCR (black bars); (**C**) – tube formation on matrigel by HCAECs in presence of 5 µg/ml ox-LDL (16 hrs); (**D**) - Graph summarizing data on matrigel angiogenesis.

Several genes (n = 31) implicated in angiogenesis were found to be significantly (p<0.05) upregulated in microarray analysis (Figure 2A) including a number of receptors and membrane-bound proteins such as NOTCH1, NOTCH4, NRP1, MMP14, KIT, PDGFR, CXCR4, TGFRSF25 and KDR (VEGFR2) as well as various components of extracellular matrix (MMP2, LAMA4, LAMA5) growth factors (PGF), cytokines (IL18), ligands (SEMA3A) and transcription factors (HES1, HES2, HEY1, HEY2, PLAU). With the exception of PLAU, all tested genes showed the same directional change as in microarray (Figure 2B). On the functional level and in agreement with earlier findings [9], presence of ox-LDL (5 µg/ml) in the culture medium stimulated tube formation on matrigel (Figure 2CD).

#### LOX-1 is involved in post-traumatic angiogenesis *in vivo*

**Figure 3:**
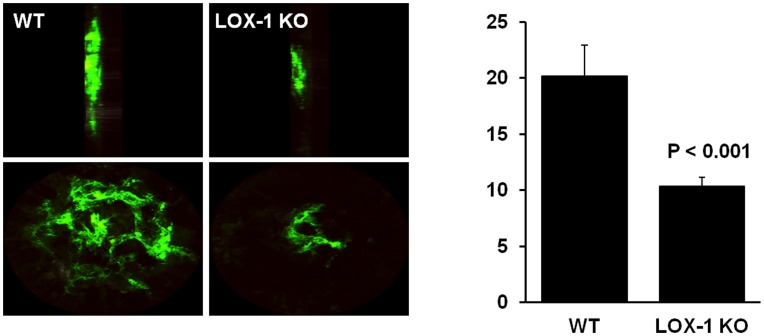
Effects of LOX-1 abrogation on choroid angiogenesis. Eyes of 8 week old wild type (C57BL) and LOX-1 KO (C57BL background) mice were subjected to laser photocoagulation (see Materials and Methods). Choroid angiogenesis was visualized in RPE-choroid-sclera flat mounts from animals perfused with 1 ml of PBS containing 50 mg/ml FITC-dextran after 7 days. Mounts were examined under a ZEISS LSM 510 laser confocal microscope and images of laser spots were captured. The images represent 3D reconstruction of the choroid neovascularization complex.

LOX-1 is a pattern recognition receptor for variety of ligands bearing oxidatively modified phospholipids [11], [12] which are to a large extent responsible for ox-LDL associated signaling including angiogenesis [13]. In order to further assess the role of LOX-1 in neovascularization *in vivo* we utilized the mouse model of chorioid neovascularization following laser photocoagulation. Seven days after injury wild type C57Bl/6 mice exhibited robust neovascularization. In marked contrast, angiogenesis in LOX-1 knockout mice comprised only about half of what was observed in wild type animals (p<0.001) (Figure 3).

### Ox-LDL Affects Both Inhibitors and Stimulators of Apoptosis

**Figure 4:**
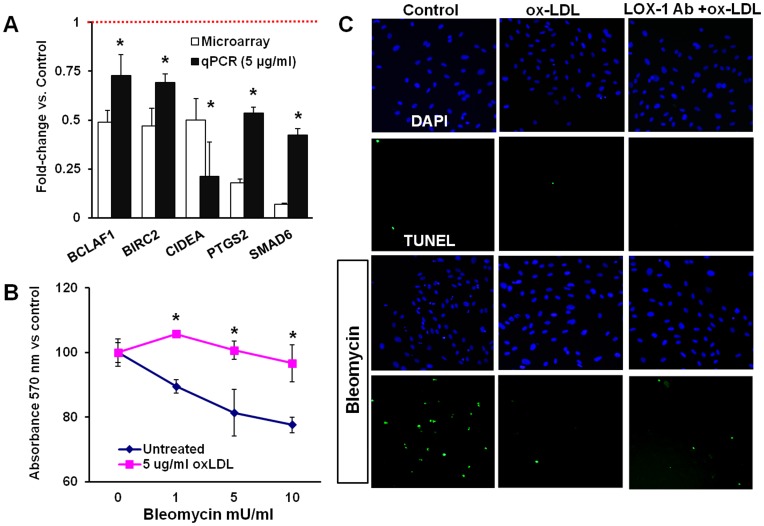
Bi-directional effects of ox-LDL on genes implicated in apoptosis. (**A**) - Differentially expressed genes involved in regulation of apoptosis (white bars) and validation of microarray data by qPCR (black bars). (**B**) –Viability of HCAECs measured by MTT assay in response to bleomycin (4 hours) in control cultures and cells pre-treated with 5 µg/ml ox-LDL for 12 hrs. (**C**) – TUNEL staining in HCAECs exposed to bleomycin (10 mU/ml for 4 hours) in control cultures and cells pre-treated with either 5 µg/ml ox-LDL or 5 µg/ml ox-LDL in presence of LOX-1 neutralizing antibody.

Among differentially expressed apoptosis related genes in response to ox-LDL treatment, we found both effectors and inhibitors of apoptosis (Figure 4A). In order to evaluate the overall impact of non-toxic concentration of ox-LDL, we measured viability of HCAECs using MTT assay after 4 hour exposure to bleomycin in control cultures and cells pre-treated with 5 µg/ml ox-LDL for 12 hours. Of note, the survival of cell subjected to 1–10 mU/ml of bleomycin for 4 hours was significantly higher in ox-LDL stimulated cells (Figure 4C). Similar trends were observed in TUNEL assay (Figure 4 B) where bleomycin had practically no impact on cells pre-treated with ox-LDL. Surprisingly, cells pre-treated with ox-LDL in presence of LOX-1 neutralizing antibody showed considerable increase in apoptosis although much lower than in control bleomycin treated cultures. This is likely indicative of internalization of ox-LDL via alternative means (passive transport or other scavenger receptors).

### Ox-LDL Transcriptome is Partially Shaped by miRNAs

**Figure 5:**
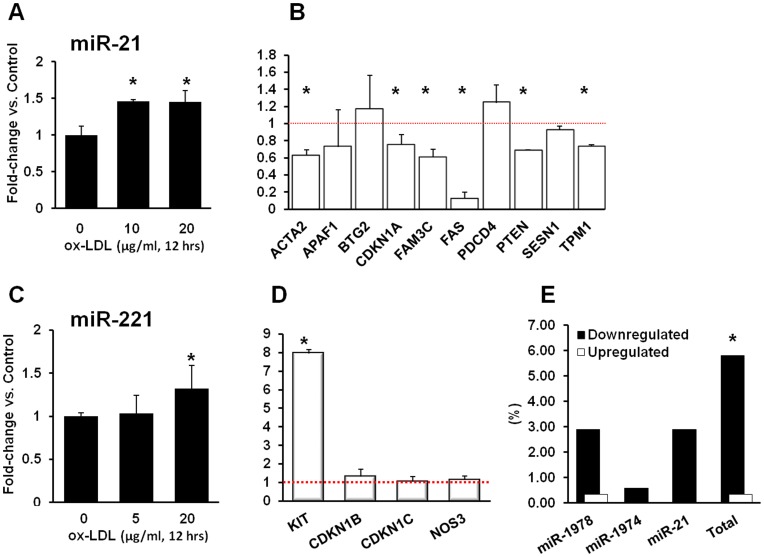
Ox-LDL signaling is partially mediated by miR-21. (**A**) - qPCR analysis of miR-21 expression in response to ox-LDL (left) and (**B**) -microarray expression of corresponding validated target genes; (**C**) - qPCR analysis of miR-221 expression in response to ox-LDL and (**D**) - microarray expression of corresponding validated target genes. (**E**) – the percentage of target genes for miR-1974, miR-1978 and miR-21 in downregulated and upregulated portions of ox-LDL induced transcriptome.

Only miR-1974 and miR-1978 were significantly upregulated by low concentration of ox-LDL (2.7-fold, p<0.02 and 2.3 fold, p<0.007 respectively, Table 2). We selected two additional miRNAs, miR-21 and miR-221 for qPCR validation as these miRNAs exhibited significant stimulation (2.7-fold and 5.7-fold respectively) and are known to be implicated in regulation of apoptosis/proliferation and angiogenesis with well-defined target genes. MiR-21 was confirmed to be stimulated by 5 µg/ml ox-LDL by 46% (p<0.03) with no further increase at 20 µg/ml (45%, p<0.05, Figure 5A). Consistent with our expectations, most miR-21 target genes were inhibited in the microarray dataset (Figure 5B). In contrast with microarray data, the expression of miR-221 was not stimulated by exposure to low concentration ox-LDL, but rose by 46% (p>0.05) when cells were exposed to 20 µg/ml ox-LDL. In line with qPCR data, miR-221 target genes were not downregulated in microarray dataset (Figure 5CD).

**Table 2:** Differentially expressed microRNAs.

	∼Fold change vs. Control	P value
miR-1978	2.34	0.0067
miR-1974	2.68	0.016
miR-221	5.72	0.132
miR-886	3.19	0.216
miR-21	2.74	0.438

In search of another measure of miRNA involvement in ox-LDL mediated expression shifts, we analyzed the percentage of target genes of miRNAs in question in downregulated and upregulated sub-groups of differentially expressed genes (Figure 5E). In downregulated set, genes targeted by miR-1974, miR-1978 and miR-21 comprised 5.8% whereas they were practically absent from upregulated part of the transcriptome (0.3%, p<0.0003).

## Discussion

In this study, we have shown for the first time that physiologic concentration of ox-LDL (5 µg/ml) evokes significant transcriptional response in human endothelial cells. Previous reports [4], [5] on ox-LDL-induced transcriptome were based on much higher concentrations (30–50 µg/ml) which have been shown to reduce the viability of vascular cells and induce apoptosis – [7], [8]. We, therefore, reasoned that selected concentration of 5 µg/ml is a more realistic approximation of the environment observed in dyslipidemia.

The wide range of transcriptional shifts involving almost 1500 genes likely reflects a signaling mosaic triggered by a multitude of lipid oxidation species found in ox-LDL. Previous reports from our group and others have shown that LOX-1 significantly contributes to all critical steps of atherogenesis and its abrogation drastically attenuates development of atherosclerosis in animal models [1], [14]. In the present study, qPCR analysis confirmed earlier findings that LOX-1 is the predominant scavenger receptor for ox-LDL in HCAECs [15]. Also, across-the-board attenuation of transcriptional response to ox-LDL in presence of LOX-1 neutralizing antibody strongly supports the critical role of LOX-1 in at least some aspects of atherogenesis involving endothelial dysfunction.

Low concentrations of ox-LDL have been reported to be angiogenic [9], [13] although not all reports are consistent with this notion [16]. Earlier studies showed that ox-LDL stimulates angiogenesis via PI3K/Akt/eNOS pathway [9] and upregulation of VEGF [17]. These effects may be assigned to specific oxidation products including 9-HODE, 13-HODE and one of the most common oxidized phospholipids ox-PAPC [13], [17].

**Figure 6:**
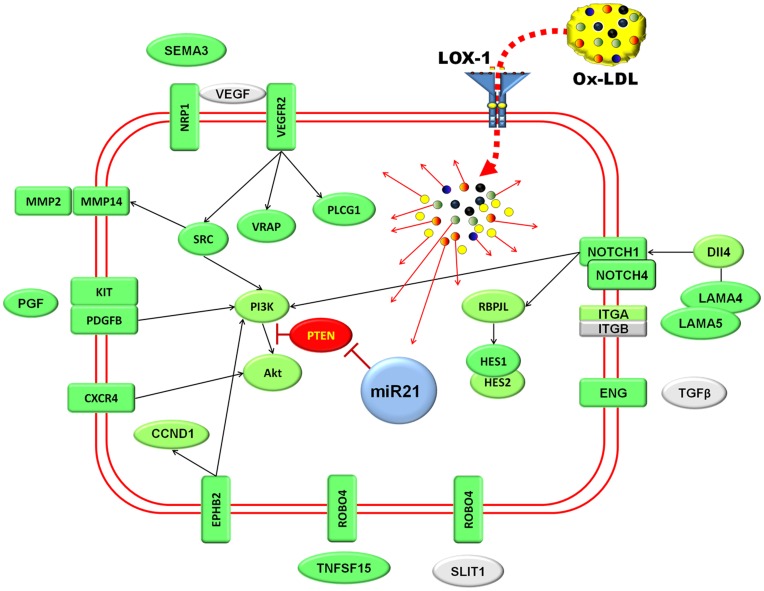
Schematics of pro-angiogenic signaling of ox-LDL based on microarray data partially validated by qPCR. Color coding: Shades of green – upregulation. Rred-inhibition; Grey –no change; Pro-angiogenic action of ox-LDL appears to be most consistently associated with VEGF and Notch pathways. Downstream target genes of VEGF also show corresponding shifts in expression patterns.

Our data indicate that ox-LDL enhances expression of multiple receptors involved in transmission of pro-angiogenic signaling from growth factors, cytokines, chemokines and components of extracellular matrix (Figure 6). Interestingly, although certain evidence of activity was found for VEGFR2 and Notch mediated signaling, upregulation of several receptors (f.e. CXCR4, EPHB2 and ROBO4) was not accompanied with increase in production of corresponding ligands or downstream transcriptional changes in target genes. This suggests that these receptors – despite their upregulation – do not necessarily contribute to pro-angiogenic effects of ox-LDL *in vitro*, although they may have a role in *in vivo* setting.

VEGFR2 - a predominant receptor implicated in various aspects of angiogenesis including proliferation, migration and survival [18] – has been stimulated 2.4 fold and, although we have not found the evidence of endogenous production of VEGF isoforms, the analysis of microarray data revealed upregulation of components of VEGF pathway that are implicated in its mitogenic action including PLCG1 and SRC. Contrary to expectations, however, CDC42 was significantly inhibited. This could be attributed to miRNA mediated inhibition as CDC42 is a putative target for miR-1974 which we found to be stimulated by ox-LDL in present study.

Notch receptors are essential for proper angiogenesis and perform dual function as a mechanism ensuring correct architecture of vascular networks and a checkpoint limiting the extent and duration of VEGF signaling by dowregulation of VEGFR2 [19]. Upregulation of Notch1 and Notch4 in ox-LDL-treated cells is likely a result of multi-fold upregulation of Delta-like ligand 4 (Dll4, p = 0.08) which could be stimulated by VEGF [20] and Laminin α4 [21]. It was accompanied with stimulation of HES1 and HES2, but inhibition of Hey1 and Hey 2 - which is a departure from usually observed unidirectional changes in expression of these effectors. Interestingly, ox-LDL also stimulated expression of endothelial cells-specific EGFL7 that has been shown to be a powerful modulator of Notch signaling [22]. EGFL7 competes with Jagged type canonical Notch ligands and thus prevents Jagged mediated signaling and, as a regulatory molecule, is critically involved in the process of tubulogenesis. The latter notion is currently under revision, as co-expressed miR-126 located within the gene sequence appears to be responsible for severe vascular deficiency phenotype that has been reported for EGFL7 deletion [23].

We identified both stimulators and inhibitors of apoptosis among genes downregulated in cells treated with low concentration of ox-LDL. BCLAF1 interacts with anti-apoptotic Bcl2 family members and promotes apoptosis upon overexpression [24]. As a component of TNF receptor signaling complex, BIRC2 promotes survival of endothelial cells and its absence leads to vascular regression due to activation of caspase-8 apoptotic program [25]. CIDEA has been found to stimulate apoptosis when over-expressed [26] although its role under physiological conditions is not clear. Interestingly, CIDE proteins have been shown to be active modulators of lipid metabolism, and their absence results in lean phenotypes, a reduction of lipid droplet size in white adipose tissue and increased metabolic rate [27]. SMAD6 is a negative regulator of TGFβ signaling [28] and its reduced expression is associated with impaired survival [29]. Presence of such a mixture of mutually exclusive trends in response to ox-LDL challenge suggests a mechanism by which overall balance may easily shift in favor of apoptosis when concentration of ox-LDL exceeds a certain threshold.

Microarray and qPCR analyses reveal upregulation of miR-1978, miR-1974 and miR-21. MiR1974 and miR-1978 are relative enigmas. Recently, these miRNAs were found to be enriched in mitochondrial RNA with putative targeting of a number of of tRNAs, rRNAs and mtDNA-coded genes [30] although significance of this phenomenon is yet to be determined. It has been shown, however, that high fat diet is associated with reduction of mitochondrial mass and impairment of functions, including inhibition of oxidative phosphorylation and biogenesis in myocytes with transcriptional changes involving primarily Complex I genes [31]–[33]. Similarly, ox-LDL causes reduction of oxygen consumption, activity of mitochondrial complexes I-IV and membrane potential in mitochondria of endothelial cells [33] that potentially can be attributed to these miRNAs.

MiR-21, on the other hand, is extensively studied and is known to be intimately involved in vascular cell physiology. MiR-21 is induced by shear stress [34], over-expressed in the vascular wall after balloon injury (where it is shown to stimulate proliferation and inhibit apoptosis) and its knockdown results in decrease in neointima formation after angioplasty *in vivo* and inhibition of proliferation in combination with increased apoptosis in vascular smooth muscle cell *in vitro* [35]. MiR-21 has been also found to be upregulated during ischemic pre-conditioning and be partially responsible for protective effect of ischemic preconditioning against cardiac ischemia/reperfusion injury [36]. The effects of miR21 are attributed to its primary target PTEN (phosphatase and tensin homolog deleted from chromosome ten). PTEN antagonizes PI3K by cleaving its major product, lipid PtdIns (3,4,5)*P_3_*, and thus preventing activation of downstream Akt signaling cascade. PTEN and other target genes for miR21 were suppressed in our microarray dataset. In addition, inhibition of PTEN should be accompanied with activation of Akt and, indeed, in our microarray dataset we observed more than 35% increase in Akt mRNA on the background of 56% upregulation of PI3K (p>0.05).

In addition, there are indirect indications that ox-LDL may trigger production of endothelium-specific miR-126. MiR-126 is positioned within exon 7 of EGFL7. Although utilized microarray chip did not contain probes for miR-126, significant upregulation of EGFL7 and downregulation of validated miR-126 target gene Spred1 (1.8-fold, p<0.002) suggests this possibility.

As mentioned earlier and shown in Figure 1C, almost 1500 genes were altered by treatment of cells with ox-LDL, many of these genes unrelated to cell survival, apoptosis or angiogenesis. In this study, we have focused on this analysis on genes that are known to affect cell survival pathway. For example, we have also identified several genes implicated in lipid metabolism and reverse cholesterol transport and other functions (not shown) which will be subject of further analysis.

It should also be noted that miRNAs can be introduced into the cells with ox-LDL particles. Recent analysis has shown that lipoproteins harbor miRNAs and that development of atherosclerosis is associated with significant changes in particles’ miRNA profiles [37]. Of relevance to present study, LDL has been found to contains excess of miR-21 (miR-1974 and miR-1978 were not analyzed). This mechanism could be one of the contributing factors, although it is not known how the process of oxidation affects stability of miRNAs in commercial ox-LDL preparations.

The ox-LDL transcriptional signature observed in the present study shows significant overlaps with microarray profiles observed with much higher concentrations of ox-LDL. Of particular interest is a study by Mattaliano et al (8) where involvement of LOX-1 in ox-LDL mediated signaling was analyzed (opposite to our approach) via overexpression of LOX-1 in endothelial cells. Both datasets exhibited significant similarities in preferentially modulated pathways including PDGF, EGF, protein kinases, cytoskeleton, migration and axonal guidance (see Table 1 and Figure S1).

In summary, our analysis shows that low concentrations of ox-LDL can trigger extensive transcriptional changes in endothelial cells that are mediated by the expression of LOX-1 and partially shaped by miRNAs. With regard to angiogenesis, oxLDL upregulates multiple receptors involved in pro-angiogenic signaling with or without accompanying ligands. We conclude that ox-LDL at low concentrations is an angiogenic factor that acts, in part, via activation of receptor network and consequent development of hypersensitivity of endothelial cells to angiogenic stimuli (Figure 6).

## Materials and Methods

### Cells and Reagents

HCAECs and corresponding vascular endothelial growth medium complemented with VEGF endothelial cell growth kit were purchased from the American Tissue Culture Collection (ATCC, Manassas, VA). High T-bar oxidized-LDL (64.2 nmoles MDA/mg protein) was purchased from Biomedical Technologies (Stoughton, MA). Antibody to human LOX-1 was obtained from Prof. T. Sawamura (National Cardiovascular Center, Osaka, Japan).

### Microarray and Pathway Analysis

Total RNA samples were purified using the RNease Kit (Qiagen) and quantified with a Ribogreen fluorescence assay on a Biotek Synergy 4 PlateReader. Quality of total RNA was assessed using an Agilent TotalRNA 6000 NanoKit on the Agilent 2100 Bioanalyzer. The Illumina TotalPrep RNA amplification kit was used to prepare biotinylated antisense RNA by starting with 500 ng totalRNA, incubating 14 hours at 37°C, and elution from a column into 100 µl of nuclease-free H_2_O. Quality and quantity of the cRNA was determined by Ribogreen fluorescence and Agilent Bioanalyzer electropherograms. Then, 750 ng of cRNA (in a total volume of 5 µl) was loaded onto the Illumina Human HT-12 v4 Gene Expression BeadChip for hybridization (17 hours @ 58°C). After blocking, staining, and washing of the microarrays, they were scanned on the Illumina iScan; data was imported into GenomeStudio v2010.3 for analysis by the Gene Expression v1.8.0 module. Internal microarray quality control data was assessed for performance of BeadChip and experiment; a purchased RNA quality control was included in each step of the RNA preparation, quantitation, and hybridization to microarrays. Sample quality was determined using scatterplots and hierarchical clustering. Prior to group or differential gene expression analyses, raw microarray data were subjected to background subtraction and average normalization on GenomeStudio. For differential gene expression, the Illumina Custom Error Model was selected. Gene annotation and pathway analysis was performed using the Expression Analysis Systematic Explorer (EASE, 10).

### Quantitative PCR

Total RNA was purified using the RNeasy mini kit (Qiagen, Valencia, CA), and cDNA was synthesized using the iScript cDNA synthesis kit (Bio-Rad) with 0.5 µg of total RNA according to the manufacturer's recommendations. qPCR was performed with pre-designed primers selected from PrimerBank [38] and ordered from Integrated DNA Technologies (Coralville, IA). RT qPCR was performed using the Applied Biosystems 7900 real-time PCR system. All qPCR reactions were carried out in a final volume of 15 µl containing 1X of SYBR Green PCR Master Mix (Applied Biosystems, Carlsbad, CA), 300 nM of each gene specific primers, 100 ng cDNA, in sterile deionized water. The standard cycling condition was 50°C for 2 min, 90°C for 10 min, followed by 40 cycles of 95°C for 15 s and 62°C for 1 min. The results were analyzed using SDS 2.3 relative quantification manager software. The comparative threshold cycles values were normalized for GAPDH reference genes. qPCR was performed in triplicate to ensure quantitative accuracy.

### MicroRNA qPCR

Primers for RT-PCR reaction and qPCR analysis were purchased from Applied Biosystems (Foster City, CA), and analysis was performed in triplicates for each data point according to manufacturer’s instructions.

### Viability Assessment

The evaluation of cytotoxicity was performed using MTT assay (ATCC) based on reduction of yellow MTT (3-(4,5-Dimethylthiazol-2-yl)-2,5-diphenyltetrazolium bromide) to purple formazan in the metabolically active mitochondria of living cells. HCAECs were seeded into 96-well plates and allowed to reach 100% confluence. Confluent cultures were exposed to various concentrations of ox-LDL for 12 hours. Upon completion of exposure, growth medium was replaced and MTT (final concentration 5 mg/ml) added. After full development of color, formazan was solubilized and absorbance was measured at 570 nm.

### Apoptosis

Apoptosis was measured by TUNEL assay using DeadEnd fluorometric TUNEL system from Promega (Madison, WI) according to the manufacturer’s instructions.

### Angiogenesis Assay

#### In vitro

50 µl of matrigel basement membrane matrix (BD Biosciences, San Jose, CA) was pipetted into each well of a 96-well plate and allowed to solidify for 30 min at 37°C. 3 × 10^4^ HUVECs in 200 µl of complete growth medium with or without ox-LDL were seeded into the wells. After 16 hrs of incubation, cells were loaded with 10 µM calcein AM (Invitrogen, Carlsbad, California, USA),), washed with PBS (x 3), and imaged using fluorescence microscopy. The area and perimeter of formed tubes was calculated using SlideBook 4.2 software (Olympus).

#### In vivo

Choroid neovascularization was studied in eight-week-old male C57BL and LOX-1 knockout mice with C57BL background (see ref. 16 for preparation of LOX-1 knockout mice). Both eyes were subjected to laser photocoagulation with an Argon laser (50-µm spot size; 4 spots close to the optic disc per eye; 0.05-s duration; 260 milliwatt) as described previously [39]. Production of a vaporization bubble at the time of laser treatment confirmed the rupture of Bruch's membrane. After 7 days, the animals were anesthetized with ketamine/xylazine mixture and perfused with 1 ml of PBS containing 50 mg/ml FITC-dextran (Sigma-Aldrich). Eyes were harvested and fixed in 10% phosphate-buffered formalin for 4 h, and retinal pigment epithelium (RPE)-choroid-sclera flat mounts were prepared. RPE-choroid-sclera flat mounts were examined under a ZEISS LSM 510 laser confocal microscope, and images of laser spots were captured. The *green* color in the laser spot represents the CNV complex. Median intensity of green fluorescence was measured using the NIH ImageJ program.

### Statistical Analysis

Data are presented as means ± standard deviation (SD). The statistical analysis was performed with SPSS 11.5 software. Multiple comparisons were analyzed by one-way ANOVA. A p value <0.05 was considered to be significant.

## Supporting Information

Figure S1 **Pathway analysis for LOX-1 antibody sensitive upregulated and downregulated genes (≥1.5-fold difference, p<0.05) that were defined as those that reversed to control values by more than ≥30%.**
(TIF)

Table S1 **List of differentially expressed genes (≥2-fold difference, p<0.01).**
(XLS)

Table S2 **List of differentially expressed genes (≥1.5-fold difference, p<0.05).**
(XLS)

Table S3 **List of LOX-1 antibody sensitive upregulated and downregulated genes (≥1.5-fold difference, p<0.05) that were defined as those that reversed to control values by more than ≥30%.**
(XLS)
