# Supplementary figures and images for: Large Impact of Low Concentration Oxidized LDL on Angiogenic Potential of Human Endothelial Cells: A Microarray Study
Source: PLoS One. 2012 Oct 24;7(10):e47421. doi: 10.1371/journal.pone.0047421 (PMC3480370; doi:10.1371/journal.pone.0047421)

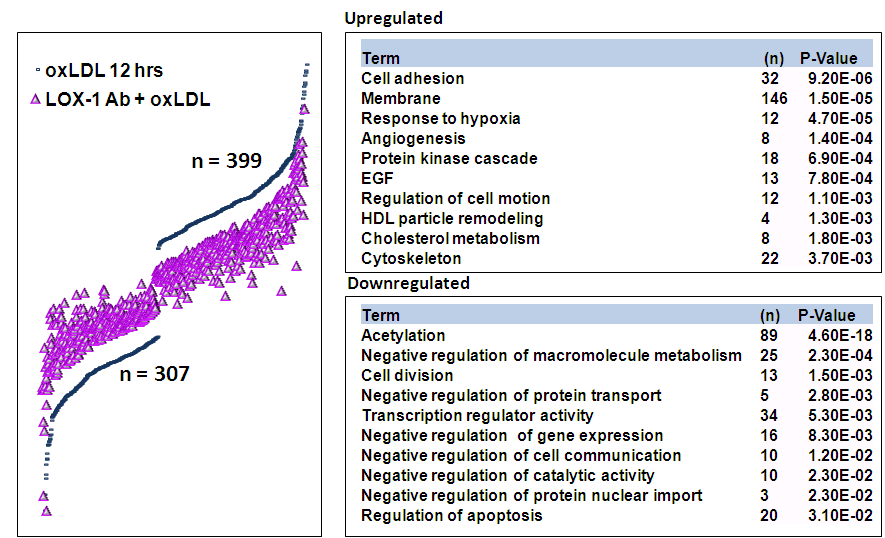

Supplement: Figure S1 — Pathway analysis for LOX-1 antibody sensitive upregulated and downregulated genes (≥1.5-fold difference, p<0.05) that were defined as those that reversed to control values by more than ≥30%. (TIF) [file pone.0047421.s001.tif]
